# Design of Block‐Copolymer Nanoporous Membranes for Robust and Safer Lithium‐Ion Battery Separators

**DOI:** 10.1002/advs.202003096

**Published:** 2021-02-18

**Authors:** Hao Yang, Xiansong Shi, Shiyong Chu, Zongping Shao, Yong Wang

**Affiliations:** ^1^ State Key Laboratory of Materials‐Oriented Chemical Engineering College of Chemical Engineering Nanjing Tech University Nanjing Jiangsu 211816 P. R. China; ^2^ Present address: College of Chemistry & Chemical Engineering Yantai University Yantai Shandong 264005 P. R. China

**Keywords:** block copolymers, lithium‐ion batteries, selective swelling, battery separators

## Abstract

Lithium‐ion batteries (LIBs) suffer from unsatisfied performance and safety risks mainly because of the separators. Herein, a block copolymer (BCP) composed of robust and electrolyte‐affinitive polysulfone (PSF) and Li^+^‐affinitive polyethylene glycol (PEG) is rationally designed to prepare a new type of LIB separator. The copolymer is subjected to selective swelling, producing nanoporous membranes with PEG chains enriched along the pore walls. Intriguingly, when used as LIB separators, thus‐produced BCP membranes efficiently integrate the merits of both PSF and PEG chains, endowing the separators thermal resistance as high as 150 °C and excellent wettability. Importantly, the nanoporous separator is able to close the pores with a temperature of 125 °C, offering the battery a thermal shutdown function. The membrane exhibits ultrahigh electrolyte uptake up to 501% and a prominent ionic conductivity of 10.1 mS cm^−1^ at room temperature. Batteries assembled with these membranes show excellent discharge capacity and *C*‐rate performance, outperforming batteries assembled from other separators including the extensively used Celgard 2400. This study demonstrates a facile strategy, selective swelling of block copolymer, to engineer high‐performance and safer LIB separators, which is also applicable to produce advanced copolymer‐based separators for other types of batteries.

## Graphical abstract

Selective swelling of polysulfone‐*block*‐polyethylene glycol leads to the production of high‐performance battery separators. Thus‐prepared separators exhibit excellent wettability, electrolyte uptake, and thermal resistance, endowing a battery with superior electrochemical performance.

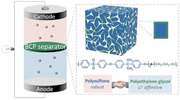

In recent years, lithium‐ion batteries (LIBs) have received extensive attention as energy storage systems such as portable electronic devices and electrical vehicles due to the high energy density, long cycle life, and high efficiency.^[^ ^1^ ^]^ The separators physically insulate the anode and cathode to prevent short circuits, while allowing Li^+^ to free flow through the pores of separators. Thus, they are a key element in LIBs and have a huge effect on the battery performance.^[^ ^2^, ^3^ ^]^

Polyolefin‐based macroporous membranes have been widely used as LIB separators owning to their good mechanical strength and acceptable electrochemical stability.^[^ ^4^ ^]^ However, the intrinsic drawbacks of polyolefin‐based separators, such as poor liquid electrolyte wettability/uptake and low thermal stability, greatly limit the further development of LIBs.^[^ ^5^ ^]^ Specifically, polyolefin‐based separators have an indifferent compatibility with liquid electrolytes and electrodes due to low polarity and surface energy. The low liquid electrolyte wettability/uptake will restrict the transport of Li^+^ and decrease charge/discharge performance of LIBs.^[^ ^6^ ^]^ The weak thermal stability of polyolefin separators would result in internal short circuit at high temperatures and cause safety problems.^[^ ^7^ ^]^ To solve these issues, other polymeric materials, such as polysulfone (PSF),^[^ ^8^ ^]^ poly(vinylidene fluoride) (PVDF),^[^ ^9^ ^]^ poly (methyl methacrylate) (PMMA),^[^ ^10^ ^]^ and polyacrylonitrile (PAN),^[^ ^11^ ^]^ have been developed to produce high‐performance separators for LIBs.

Among above‐mentioned candidates, PSF is considered as an excellent precursor that can be adopted for the preparation of battery separators due to its high thermal stability, chemical resistance, mechanical strength, and easy processability. More importantly, PSF has a good affinity with liquid electrolytes as it is enriched with C—O—C and O=S=O functional groups. Cheng et al. prepared PVDF/PSF blend membranes of polymer electrolyte for LIBs.^[^ ^8^ ^]^ The addition of PSF significantly increased the electrochemical stability and improved charge‐discharge capacity compared with the bare PVDF membrane. Unfortunately, few researchers have been devoted to fabricate PSF‐based separators.

Apart from PSF, hydrophilic polyethylene glycol (PEG) (high molecular weight of polyethylene glycol called PEO) has been largely studied as a positive modifier to enhance the performance of LIB separators, owing to its excellent affinity with liquid electrolyte and the ability to generate complexes with lithium salts.^[^ ^12^ ^]^ The ether oxygen atoms in PEG tend to interact with Li^+^ and promote the dissolution of lithium salts, facilitating the Li^+^ transport by the segmental motion of PEG chains. For example, Li et al. successfully designed novel “active separators” by incorporating cross‐linked PEG with commercialized polypropylene (PP) separators, which could effectively improve the stability of liquid electrolyte uptake, ionic conductivity and electrochemically stable window.^[^ ^13^ ^]^ Kim et al.^[^ ^14^ ^]^ and Liang et al.^[^ ^15^ ^]^ prepared LIB separators by coating PEO onto microporous polyethylene (PE) and polyimide (PI) membranes, realizing improved battery performances. The electrolyte uptake rate of PEO‐coated PI separators was higher than PP separators and PEO‐coated PI separator had the highest ionic conductivity of 3.83 mS cm^−1^, which was greatly higher than PI and PP separators (1.87 and 0.29 mS cm^−1^). Though some effective methods to produce PEG‐incorporated separators have been reported, improving the stability of PEG in these matrix still remains a huge challenge as the loss of unstably‐blended PEG could occur during charge/discharge processes, which will inevitably lead to decreased battery performances.

To fully take the advantages of PSF and PEG in LIB separators, block copolymer (BCP) of polysulfone‐*block*‐polyethylene glycol (PSF‐*b*‐PEG, SFEG) comes into sight. BCPs are composed of two or more thermodynamically incompatible homopolymer chains linked by stable covalent bonds, and phase separation in microscale can occur in BCPs, that is “microphase separation.”^[^ ^16^, ^17^ ^]^ The phase‐separated BCP films can be nondestructively converted into nanoporous membranes by selective swelling‐induced pore‐making process.^[^ ^18^, ^19^, ^20^, ^21^ ^]^ In our previous studies, SFEG was utilized as raw materials to produce ultrafiltration membranes by selective swelling and successfully obtained membranes with excellent mechanical strength, antifouling property, and permselectivity.^[^ ^22^ ^]^ Given Li^+^‐affinitive PEG chains will migrate and enrich on pore walls after swelling, highly cavitated SFEG membranes are highly promising candidates for the construction of advanced battery separators. Besides, the strong covalent bonds between PSF and PEG guarantee the stability of PEG so as to prevent the loss of PEG, giving a stable and high battery performance. The PSF matrix that holds robust and electrolyte‐affinitive benefits will also provide the mechanical stability and enhance separator performances.

Herein, we demonstrate a facile approach, namely selective swelling, to prepare SFEG membranes that were used as separators for high‐performance LIBs. By combining the advantages of PSF and PEG, thus‐produced SFEG membranes showed excellent thermal stability, better wettability, higher liquid electrolyte uptake, and ionic conductivity due to the unique physicochemical structure of SFEG (**Figure** **1**a). Such a novel SFEG membrane would return to its initial dense structure at 125 °C, which promises a thermal shutdown function to enhance battery security. Furthermore, LIBs assembled with SFEG membranes exhibit excellent cycle stability and *C*‐rate performance, outperforming the performance resulted from commercial Celgard 2400 separators.

**Figure 1:**
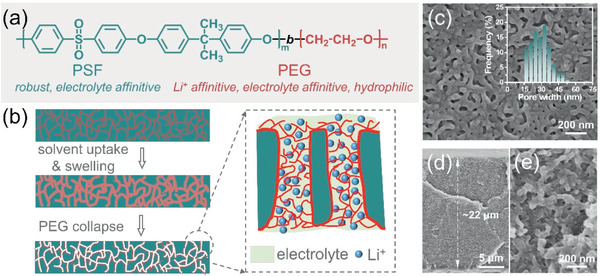
Selective swelling of SFEG membranes. a) Molecular structure. b) Illustration for selective swelling of SFEG and its application as the LIB separator. c) Surface and d,e) cross‐sectional morphologies of SFEG membranes. Inset in (c) presents the pore width distribution of SFEG membranes.

The dense SFEG self‐supporting films were obtained by knife‐coating on a clean glass followed by drying. Selective swelling‐induced pore generation, which can be divided into three steps: uptake of swelling agents, swelling of SFEG, and drying with solvent evaporation, was then adopted to cavitate dense SFEG films by simply soaking them into the acetone/*n*‐propanol mixture.^[^ ^23^, ^24^, ^25^ ^]^ Notably, the PEG cylindrical phase is randomly distributed in the PSF matrix after dense film formation. Here, the mixture of acetone and *n*‐propanol is deliberately selected as the swelling agent. Particularly, *n*‐propanol possesses strong affinity with PEG chains and acetone gives a promotion on the plastic deformation of PSF matrix. Upon the immersion of the SFEG film into swelling agent, solvent molecules are preferentially enriched in the PEG cylindrical phase due to the better affinity with PEG and expand PEG's volume. The expanded PEG phase will squeeze PSF matrix and the deformation of PSF inevitably occurs. Further, the expanded PEG phase will connect and merge with their neighbors, forming a continuous PEG phase distributed in PSF matrix. After removing SFEG films from the swelling agent, the swollen PEG chains collapse and deformed PSF chains congeal with the fast evaporation of solvents, as shown in Figure 1b. Thus, nanopores are generated along the position initially occupied by expanded PEG phase, and nanoporous SFEG membranes are obtained. The nondestructive advantage of selective swelling can be perceived as the PEG chains are not dissolved or etched away, instead attaching to line along the pore walls, thus facilitating the infiltration of electrolyte into SFEG membranes and transport of Li^+^ (Figure 1b). Figure 1c shows the surface morphology of SFEG membranes, and we can observe a 3D interconnected porous structure consisted by circular and elongated pores. The pore width is varied from ≈15 to 50 nm, giving an average pore width of approximately 30 nm based on the diameter of circular pores and the width of elongate channels. Thus‐produced SFEG membrane possesses a thickness of ≈22 µm, and the magnified cross‐sectional morphology of SFEG membrane is similar to the surface one (Figure 1d,e). Moreover, the nanoporosity in SFEG membranes is uniformly distributed through the entire layer without any obstructed areas, promising an unimpeded infiltration of electrolyte and migration of Li^+^ when utilized as separators.

The composition of SFEG membranes was then confirmed by a Fourier transform infrared (FTIR) spectrophotometer. As shown in **Figure** **2**a, the spectra show peaks around 1322, 1293 cm^−1^ and 1235, 1102 cm^−1^, corresponding to sulfone and ether stretching vibrations, respectively. The spectra of the SFEG membranes before and after selective swelling give no obvious change, which demonstrates that the two blocks solidly exist in the process of pore generation. Benefitting from both the strong covalent bonds and inherent thermal stability of two polymeric chains, the SFEG membrane exhibits a high thermal stability of up to 380 °C, and the thermal stability is comparable to Celgard 2400 (Figure 2b). Further, the tensile strength of the SFEG membrane is as high as 14.8 MPa, showing appropriate mechanical strength for serving as the separator (Figure 2c). Contact angle tests indicate that the cavitated SFEG membrane shows a pure water contact angle of ≈65° (Figure 2d), and the hydrophilicity is greatly higher than that of Celgard 2400 (≈121°). The pronounced hydrophilicity of SFEG membranes here can be ascribed to the enrichment of water‐affinitive PEG chains on the membrane surface during the selective swelling as mentioned above.

**Figure 2:**
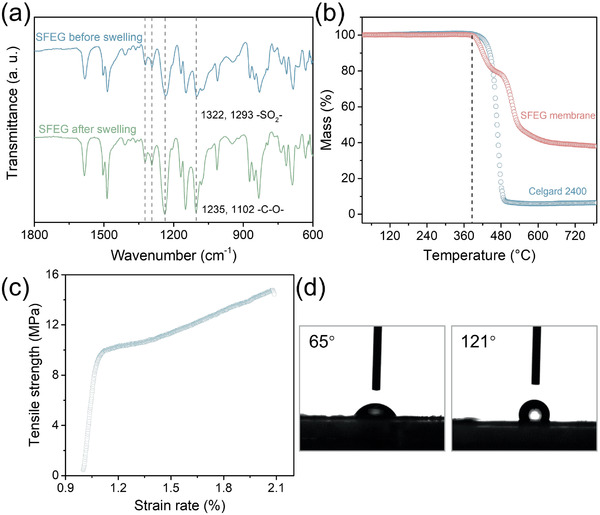
Characterizations of SFEG membranes. a) FTIR spectra of SFEG membranes before and after swelling. b) Thermogravimetric curves. c) Stress–strain curves. d) Water contact angles of the SFEG membrane (left) and Celgard 2400 (right).

The separator having satisfactory wettability and liquid electrolyte uptake can effectively retain the electrolyte, hence facilitating fast Li^+^ transport between anode and cathode during charge/discharge cycling.^[^ ^26^ ^]^ To directly evaluate the wettability, a same volume of liquid electrolyte was dropped onto the surface of SFEG membranes and Celgard 2400 separators. **Figure** **3**a presents the digital image during wettability testing. After standing for 5 s, Celgard 2400 separator can be hardly wetted and liquid electrolyte solution forms a bead on the surface (Figure 3a left). As for SFEG membranes, the liquid electrolyte is quickly infiltrated within a short duration of 5 s, indicating the excellent wettability of SFEG membranes (Figure 3a right). The quick infiltration and spread of liquid electrolyte in the case of SFEG membranes can be ascribed to the favorable affinity of liquid electrolyte to PSF and PEG. In contrast, additional surface modification to enhance the affinity of commercial polyolefin‐based separators, such as Celgard 2400, to liquid electrolyte are usually required, which would involve tedious, cumbersome pretreatment, uncontrolled reaction, and purification processes.^[^ ^27^, ^28^ ^]^ The electrolyte uptake performance was then investigated with results given in Figure 3b. The SFEG membrane is readily wetted in the liquid electrolyte within a few seconds and the electrolyte uptake is 480% after soaking for only 2 min. After immersing for 1 h, the SFEG membrane exhibits an ultrahigh electrolyte uptake of up to 501%, which is more than seven times higher than Celgard 2400 separators (67% electrolyte uptake). We should note that the SFEG membrane and Celgard 2400 separator give a similar thickness of ≈22–24 µm, eliminating the thickness‐induced uptake difference.^[^ ^2^ ^]^ Besides, the SFEG membrane and Celgard 2400 were measured to possess similar porosities (i.e., 36.9% for SFEG membrane, and 30.8% for Celgard 2400). Therefore, the pronounced electrolyte uptake of SFEG membrane can be mainly attributed to intrinsic functional groups (C—O—C and O=S=O) in SFEG. These groups possess an excellent affinity with liquid electrolyte, which contributes to facilitate the penetration of liquid electrolyte into SFEG pores. Hydrophilic PEG chains on the pore surfaces and walls of membranes may accelerate this infiltration process as well.

**Figure 3:**
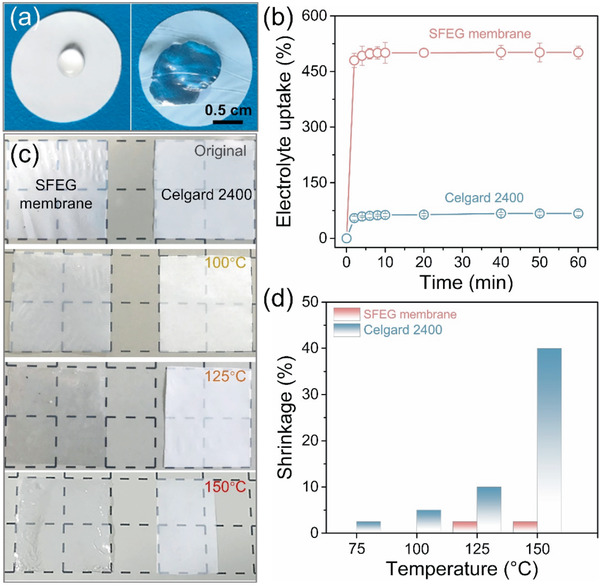
Physicochemical properties of SFEG membranes and Celgard 2400 separators. a) Digital image of the wettability. b) Electrolyte uptake performance. c) Digital images of thermal stability after heating at 100, 125, and 150 °C. d) Size shrinkage ratio.

When used as separators for LIBs, porous membranes should possess desired thermal stability to prevent heat shrinkage during the charge–discharge process, thus avoiding the safety problem caused by the short circuit between anode and cathode.^[^ ^29^ ^]^ Figure 3c shows digital images of SFEG membranes and Celgard 2400 separators after thermal treatment at different temperatures for 1 h. Obviously, the sizes of SFEG membranes remain basically unchanged at various temperatures in the range of 75–150 °C, while Celgard 2400 separators display remarkable shrinkage after heating at temperatures above 100 °C. To gain more insight, the size shrinkage ratio was then calculated by comparing the membrane area before and after heating. It is worth noting that the SFEG membrane shows negligible size change at 75 and 100 °C, and the shrinkage ratio is correspondingly considered as 0, as given in Figure 3d. On the contrary, the size shrinkage ratio for Celgard 2400 separator greatly increases from 2.5% to 40% with the temperature rising from 75 to 150 °C. Moreover, the integrity of the SFEG membrane is well maintained when subjected to the organic electrolyte with a temperature of up to 100 °C (Figure S1, Supporting Information). Thus‐produced SFEG membranes also display a durable stability in the organic electrolyte, evidenced by no structure and lithium‐ion conductivity changes after soaking in the electrolyte for 9 d (Figure S2, Supporting Information). Typically, Celgard 2400 separators are a type of single‐layer PP membranes. As the fabrication of PP separators involves a stretching procedure, the separator tends to shrink under a relatively high temperature.^[^ ^30^ ^]^ As for selective swelling‐induced SFEG membranes, the PSF matrix possesses a high glass transition temperature (*T*_g_ = 186 °C)^[^ ^31^ ^]^ and is able to overcome the size deformation, giving a negligible thermal shrinkage (2.5%) under a high temperature of up to 150 °C. These results confirm that self‐standing nanoporous SFEG membranes prepared by selective swelling are able to serve as the separator for LIBs, and hold superior thermal stability, which guarantees the battery performance when running at high temperatures. Importantly, owning to the thermal annealing, pores of the SFEG membrane can be closed under temperatures above 125 °C. For instance, the initial nanoporous structure disappears with the generation of a dense structure after treating at 125 °C for 1 h (Figure S3, Supporting Information). The color change from milky to transparent evidences the disappearance of nanopores (Figure 3c, treated at 125 °C). Notably, the pore close induced by the thermal annealing causes no influence to the size of SFEG membrane. Further, the pore close can effectively reduce the ionic conductivity and cut off the electrode reactions, offering the battery equipped with SFEG membranes a shutdown function.^[^ ^2^ ^]^ This thermal‐induced shut‐down function is further demonstrated by the reduced ionic conductivity from 0.27 to 0.04 mS cm^−1^ with the treating temperature rising from 125 to 150 °C (Figure S4, Supporting Information).

**Figure** **4**a presents Nyquist plots of SFEG membranes and Celgard 2400 separators. The intercept of inclined spike on the *Z*′ axis from the Nyquist plot is considered as the bulk resistance. The ionic conductivity of SFEG membranes and Celgard 2400 separators based on the AC impedance spectroscope was calculated by Equation (4) (see Experimental Section). Accordingly, the bulk resistance of SFEG membranes and Celgard 2400 separators are 0.7 and 11.4 ohm, respectively. Moreover, the SFEG membrane holds a high ionic conductivity of 10.1 mS cm^−1^ while Celgard 2400 separator possesses an ionic conductivity of 0.65 mS cm^−1^. As for LIBs, the ionic conductivity corresponds to the capacity and mobility of Li^+^.^[^ ^32^ ^]^ As we discussed above, the SFEG membrane has a high wettability and electrolyte uptake, revealing that the separator possesses a considerably high Li^+^ capacity. Besides, PEG chains, as one building block of SFEG, are capable of dissolving lithium salt through the cooperative interaction with Li^+^ to improve ionic conductivity. Apart from the transport of Li^+^ in the liquid electrolyte, the movement of Li^+^ is also coupled with the PEG chains enriched on the SFEG pore walls (Figure 1b).^[^ ^27^ ^]^ The high Li^+^ capacity and enhanced Li^+^ mobility simultaneously facilitate the Li^+^ conductivity, endowing the SFEG membrane‐assembled LIB with an enhanced ionic conductivity.

**Figure 4:**
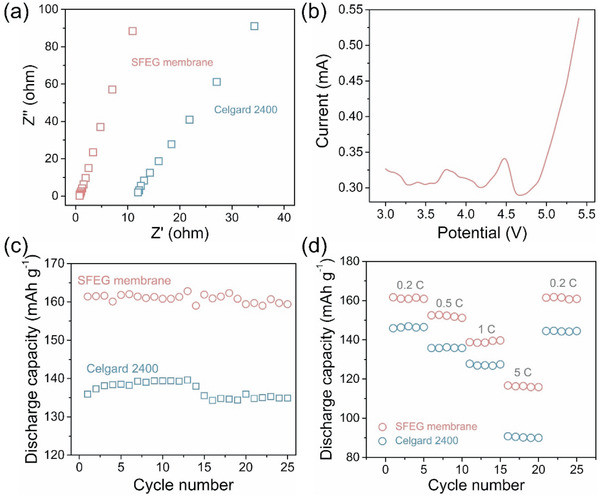
Electrochemical performances of LIBs with SFEG membranes and Celgard 2400 separators. a) AC impedance. b) Linear sweep voltammetry curve of SFEG membranes. c) Cycle charge and discharge capacity of LIBs at 0.2 C rate. d) Discharge capacity of LIBs at various charge rates.

Electrochemical stability of separators is critical to the charge/discharge stability for LIBs. Under linear sweep voltammetry measurement, the current value of cell varies in a relatively stabilized range (≈0.3–0.32 mA) with the potential rising from 3 to 4.7 V (Figure 4b). When the potential reaches 4.7 V, the current value greatly rises due to the decomposition of electrolyte.^[^ ^33^ ^]^ Therefore, the LIB equipped with a SFEG membrane can safely work at voltages ranging from ≈3 to 4.2 V, indicating the SFEG membranes served as the separator are highly robust to endure the operating voltage. Here, the excellent electrochemical stability of SFEG separators originates from high ionic conductivity and well affinity between SFEG and liquid electrolyte.^[^ ^34^ ^]^

The first charge–discharge capacities of SFEG membrane and Celgard 2400 are presented in Figure S5 in the Supporting Information. The cell based on SFEG membrane reveals the first charge and discharge capacities of 166 and 161 mA h g^−1^, respectively. In contrast, the cell based on Celgard 2400 exhibits lower charge and discharge capacities of 145 and 138 mA h g^−1^, respectively. This is due to the higher ionic conductivity of SFEG membranes since a higher ionic conductivity can promote the repeated intercalation/deintercalation of carrier ions in/from the electrode materials. To evaluate electrochemical performances, the cell consisted by a SFEG membrane and two electrodes was prepared. Figure 4c shows the discharge capacities with 25 cycles of LIBs using SFEG membranes and Celgard 2400 separators. The battery with SFEG membranes possesses a similar tendency as the battery with Celgard 2400 separators, indicating the batteries have stable charge‐discharge performance when using the SFEG membranes as separators. The cycling stability also implies that the SFEG membranes are electrochemically stable between the electrolyte and electrode materials, and there are no side reactions that would cause capacity instability. Additionally, the discharge capacity of battery with SFEG membranes is higher than the battery with Celgard 2400 separators within 25 charge–discharge cycles. This enhanced discharge capacity can be explained by the higher affinity between SFEG membranes and liquid electrolyte, which enables the electrode materials sufficiently wetted and promotes the ionic conductivity. Generally, the electrode materials play a major role in the discharge capacity of the batteries. Besides, the separator has an important influence on the capacity of battery as well because the separator as a medium can directly affect the transport of ions for the electrochemical reaction and separate two electrodes.^[^ ^35^ ^]^ A pronounced electrolyte uptake can easily and effectively wet electrode materials, contributing to the intercalation and deintercalation of LIBs on the cathode and further improving the discharge capacity.^[^ ^33^ ^]^ More importantly, the better wettability of SFEG separators helps to form a stable solid electrolyte interface layer in the coin cell, which would decrease the aggregation of Li^+^ and improve coulombic efficiency.

The rate capabilities of LIBs with SFEG membranes and Celgard 2400 separators were then measured at different *C*‐rates for 25 cycles, as given in Figure 4d. The batteries with SFEG membranes show stable charge/discharge capacity during cycling at different *C*‐rates, and the discharge capacities are 161, 152, 138, and 116 mA h g^−1^ at 0.2, 0.5, 1, and 5 C, respectively. Here, the discharge capacities gradually decrease with increased discharge current density, which reveals the energy loss caused by fast ions motion and high polarization under this condition. When the *C*‐rate is reduced to 0.2 C, the capacities of LIBs with SFEG membranes can recover to the original level, implying the stability of LiFePO_4_ materials is retained.^[^ ^36^ ^]^ Compared to Celgard 2400 separators, the LIBs with SFEG membranes always exhibit higher discharge capacities at 0.2–5 C current densities, demonstrating the battery with SFEG membranes holds a higher cathode utilization and discharge *C*‐rate capacities, matching well with the above‐mentioned merits of SFEG membranes. The robustness of SFEG membranes also promises a stable discharge capability during the long‐cycle test (Figure S6, Supporting Information).

Methods to produce polymer‐based separators is summarized in Table S1 in the Supporting Information. Obviously, selective swelling strategy is extremely simple without any tedious procedures, offering a great possibility to scale up the manufacturing process of BCP‐based membranes for separators. The main properties of polymeric separators prepared by various materials are also compared and given in **Table** **1**. Thanks to the high glass‐transition temperature of PSF, the SFEG membrane displays a relatively high thermal resistance. The membrane also has a superior affinity with liquid electrolyte, evidenced by its well wettability and high electrolyte uptake. These merits will correspondingly contribute to obtain a greatly improved ionic conductivity (Table 1) and battery performances. Therefore, these attractive advantages make the selective swelling of SFEG to be a promising route for the manufacture of separators towards high‐performance LIBs. Further, other advanced separators can be designed and prepared by selective swelling of tailored BCPs as well given the universality of this pore‐making approach.

**Table 1:** Performances of polymeric separators for LIBs

Material	Fabrication method	Thermal shrinkage [%]	Electrolyte uptake [%]	Ionic conductivity [mS cm^−1^]	Ref.
PP	Commercial	40% at 150 °C for 1 h	67	0.65	/
PE	Commercial	98% at 150 °C for 0.5 h	106	0.36	^[^ ^37^ ^]^
PVDF	Electrospinning	0% at 135 °C for 1 h	140	/	^[^ ^38^ ^]^
PVDF	TIPS	/	213	0.4	^[^ ^39^ ^]^
PVDF‐HFP^a)^	Phase inversion	18.3% at 150 °C for 1 h	125	0.24	^[^ ^40^ ^]^
PVDF‐HFP	Electrophoretic deposition	5.06% at 160 °C for 1 h	/	/	^[^ ^41^ ^]^
PEI^b)^	Solution casting	0% at 160 °C for 1 h	197	0.88	^[^ ^42^ ^]^
PAN	Electrospinning	/	363	0.94	^[^ ^43^ ^]^
PI	Electrospinning	0% at 200 °C	138.5	0.829	^[^ ^44^ ^]^
Polypyrrole	Casting through vacuum filtration	0% at 200 °C for 10 min	130	/	^[^ ^45^ ^]^
Bombyx Mori silkworm cocoons	Directly used	/	400	0.4	^[^ ^46^ ^]^
Chitin nanofibers derived from prawn shells	Solution casting	0% at 150 °C for 1 h	242	0.064	^[^ ^47^ ^]^
PAEK^c)^	Electrospinning	0% at 150 °C for 1 h	561	2.73	^[^ ^48^ ^]^
PTFE^d)^	Electrospinning	0% at 170 °C for 1 h	330	1.866	^[^ ^49^ ^]^
Cellulose	Evaporation induced self‐assembly	/	240	2.7	^[^ ^50^ ^]^
PSF‐*b*‐PEG	Selective swelling	2.5% at 150 °C for 1 h	501	10.1	This work

^a)^ Poly vinylidene fluoride‐hexafluoropropylene

^b)^ Polyetherimide

^c)^ Poly(aryl ether ketone)

^d)^ Polytetrafluoroethylene.

In summary, this work presents a new, simple, and efficient strategy to fabricate high‐performance BCP‐based membranes for LIB separators. Completely different from traditional blending approaches using homopolymers, selective swelling of BCPs not only possesses the preparation convenience but also fully integrates the virtues of two homopolymers without polymer losing. The resultant SFEG membranes exhibit excellent thermal resistance and low shrinkage with the existence of robust PSF chains. Impressively, the selective swelling‐induced pores of SFEG membranes can be closed at temperatures above 125 °C, providing thus‐assembled batteries a shutdown function. Compared with commercial polypropylene separators (Celgard 2400), the SFEG membranes possess better electrolyte wettability and remarkable electrolyte uptake of up to 510%, leading to higher ionic conductivity. Moreover, the LIBs assembled with SFEG membranes exhibit stable and excellent cycle performance and *C*‐rate capacity. These outstanding performances convincingly prove that the nanoporous SFEG membranes are capable of serving as separators for high‐performance LIBs. Our work simultaneously opens up a new avenue and a novel material platform to produce separators from block copolymers, greatly meeting the critical requirement for designing advanced separators for batteries.

## Conflict of Interest

The authors declare the following competing financial interest(s): Nanjing Tech University has filed the patent application related to the findings described in this manuscript.

## Supporting information

Supporting Information
