# Supplementary material for: Design of Block‐Copolymer Nanoporous Membranes for Robust and Safer Lithium‐Ion Battery Separators
Source: Adv Sci (Weinh). 2021 Feb 18;8(7):2003096. doi: 10.1002/advs.202003096 (PMC8025019; doi:10.1002/advs.202003096)
Supplement: Supplementary file 1 — Supporting Information [file ADVS-8-2003096-s001.pdf]

WILEY-VCH

© 2020 Wiley-VCH GmbH

ADVANCED SCIENCE
Open Access

Supporting Information

for *Adv. Sci.*, DOI: 10.1002/advs.202003096

Design of Block-Copolymer Nanoporous Membranes for Robust and Safer Lithium-ion Battery Separators

*Hao Yang, Xiansong Shi, Shiyong Chu, Zongping Shao, and Yong Wang**

Supporting Information

Design of Block-Copolymer Nanoporous Membranes for Robust and Safer Lithium-ion Battery Separators

Hao Yang[†]*, Xiansong Shi, Shiyong Chu, Zongping Shao, and Yong Wang**

State Key Laboratory of Materials-Oriented Chemical Engineering, College of Chemical Engineering, Nanjing Tech University, Nanjing 211816, Jiangsu, P. R. China
E-mail: yongwang@njtech.edu.cn

H. Y. and X. S. contributed equally to this work.

[†]Present address: College of Chemistry & Chemical Engineering, Yantai University, Yantai 264005, Shandong, P. R. China

Experimental Section

Materials

Block copolymer of polysulfone-*block*-polyethylene glycol (PSF-*b*-PEG, SFEG), with the polydispersity index (PDI) of 2.0, molecular weight (M_w) of 79.1 kDa and W(PEG)=21%, were purchased from Nanjing Bangding. Commercial polyolefin separators (polypropylene, Celgard 2400) were obtained from Celgard, LLC., USA and used for comparison. The liquid electrolyte solution containing hexafluorophosphate lithium ($LiPF_6$) (1 mol/L) and mixture solvent ethylene carbonate (EC)/dimethyl carbonate (DMC) (1:1, vol/vol) was purchased from Suzhou Dodochem Co., Ltd., China. DI water (1-5 μS/cm) was used throughout this work. All other solvents were purchased from local suppliers and used as received.

Preparation of SFEG separators

The SFEG separators were prepared by knife-coating and selective swelling process. Specifically, SFEG were dissolved in 1,2-dichloroethane with a concentration of 15 wt%. Then the solution was mechanically stirred for 4 h at room temperature to ensure polymers dissolve completely. Subsequently, 10-15 mL SFEG solution was dropped onto a clean glass and coated at a gate height of 100 μm by knife. After coating, the wet SFEG film was dried at 120°C for 5 min and separated from the glass by immersing in DI water. The SFEG membranes were then vacuum dried at 80°C for 12 h to remove residual solvent and water. Thus-produced dense SFEG self-supporting film was immersed into 60°C acetone/*n*-propanol (1:4 wt/wt) mixture solution for 4 h to generate pores. After swelling, the SFEG separator was vacuum dried at 60°C for 5 h and then stored at room temperature before further use.

Characterization

The surface and cross-sectional morphologies of SFEG separators were observed using a field-emission scanning electron microscope (SEM, Hitachi S4800, Japan). The samples were soaked into liquid nitrogen and quickly fractured to obtain cross-sectional morphology. Before examination, the samples were vacuum sputtered a thin layer of Pt/Au alloy to enhance their conductivities. A software namely NanoMeasurer was used to statistically analyze the pore size of the SFEG membranes.

The wettability of SFEG separators and Celgard 2400 separators were measured by dropping a same volume liquid electrolyte on the sample surface and compared the wetting situation of separators after 5 s. Water contact angle tests were performed on a goniometer (DropMeter A100P, Maist).

Fourier transform infrared spectrometer (FTIR, Nicolet 8700, Thermo Fisher Scientific) was applied to reveal the composition of SFEG membranes before and after selective swelling, and the test was conducted at attenuated total reflection mode. A universal testing machine (CMT-6203, MTS) was used to test the tensile strength of the SFEG membrane. Thermal

gravimetric analysis was obtained by a Netzsch STA 409PC thermal analyzer with the temperature ranging from 25 to 800°C under a heating rate of 10°C min^{-1} in N_2.

The porosity (ε) of SFEG membranes and Celgard 2400 was calculated by the following equation:

$$\varepsilon=(W_1-W_0)/(\rho Al) \qquad (1)$$

where ρ (g cm^{-3}) is the density of pure water, A (cm^2) is the membrane area, and l (cm) is the membrane thickness. W_1 and W_0 are the weight of the membrane before and after saturating in pure water. Please note that before weighing the excessive water on the membrane surface is carefully removed after soaking in water for a sufficient duration.

The liquid electrolyte uptake of separators was measured by the weight difference of separators before and after liquid electrolyte soaking for a certain time. The dried mass of SFEG separators and Ceglard 2400 separators were measured with a separator size of 20 mm × 20 mm. The test process was carried out for 1 h with continuous recording the mass change of separators in the glove box (Super, Mikrouna, China). Before weighing, the redundant electrolyte solution on the surface of separators was absorbed with filter paper. The liquid electrolyte uptake of separators was calculated by the following equation:

$$P=(W-W_0)/W_0\times 100\% \qquad (2)$$

where P (%) is the electrolyte uptake of separators, W_0 (g) is the weight of dry separators and W (g) is the weight of wet separators after soaking into liquid electrolyte.

The thermal shrinkage of SFEG separators and Celgrad 2400 separators was measured by heating at different temperatures (25, 50, 75, 100, 125 and 150°C) for 1 h. The separators were fixed on the glass slide to prevent the curl of separators during heating process. The thermal shrinkage is calculated by the following equation:

$$\eta=(S-S_0)/S_0\times 100\% \qquad (3)$$

where η (%) is the thermal shrinkage of separators, S (m^2) and S_0 (m^2) are the area of separators before and after heating.

The ionic conductivity of the SFEG separators and Celgard 2400 separators was measured by AC impedance measurement using an electrochemical workstation (CHI 604D, Shanghai Chenhua Apparatus, China). The separator was sandwiched between two stainless steel (SS) blocking electrodes and assembled in a CR2032 coin cell. The impedance was measured in the frequency ranging from 1 Hz to 10^5 Hz at an amplitude of 5 mV. The ionic conductivity of separators was calculated by the following equation:

$$\sigma=d/(R\times S) \qquad (4)$$

where σ (S/cm) is the ionic conductivity, R (ohm) is the bulk resistance, d (μm) and S (m^2) are the thickness and effective area of the separators, respectively.

The electrochemical stability of the SFEG separators and Celgard 2400 separators was examined through linear sweep voltammeter (LSV) in a cell of lithium foil/separators/SS at the scanning rate of 2 mV/s over the potential ranging from 3 to 5.5 V.

To measure the electrochemical performances, the prototype lithium-ion batteries were assembled through sandwiching fabricated SFEG separators (or Celgard 2400 separators) between $LiFePO_4$ cathode and lithium metal anode in CR2032 coin cells. Previous to assemble cells, separators were dried at 70°C for 24 h under vacuum to remove water absorbed in separators. All cells were assembled inside argon-filled glove box with oxygen and water content <0.1 ppm to avoid absorbing water. Charge-discharge cycle performance was tested at room temperature in a battery test system (LAND CT2001A, Wuhan Lanhe, China) between 2.5 V and 4.2 V at 0.2 C rate. The *C*-rate performance of lithium-ion batteries with SFEG separators/Celgard 2400 separators was measured in a potential range of 2.5-4.2 V at different current densities (0.2, 0.5, 1.0 and 5.0 C). The long-cycle test was also performed by using the SFEG membrane prepared with a gate height of 200 μm.

Figures and Tables

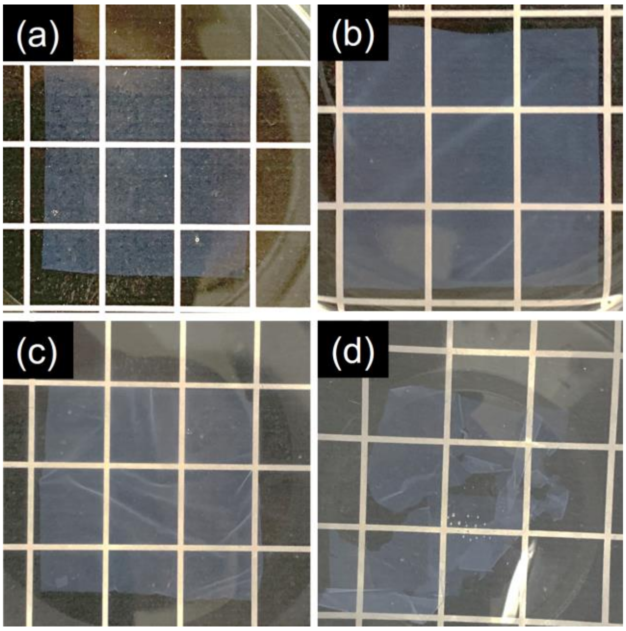

Figure S1. Digital images of the SFEG membranes after soaking in hot electrolyte with a temperature of 25°C (a), 75°C (b), 100°C (c), and 125°C (d) for 1 h.

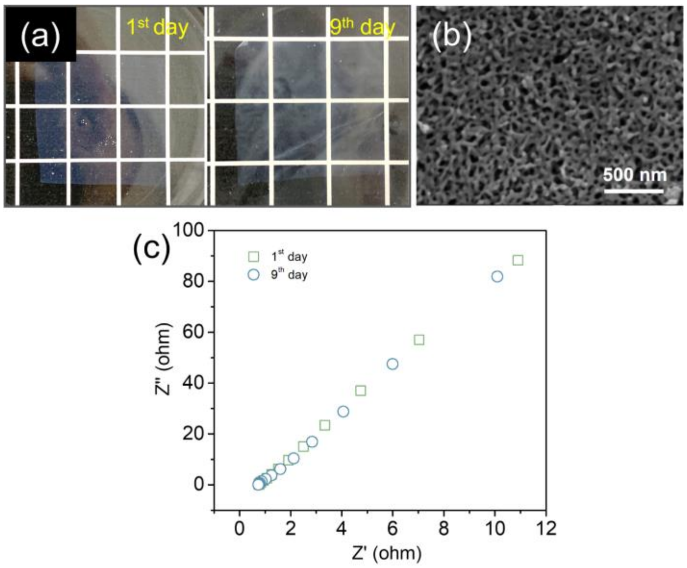

Figure S2. Digital images (a), SEM image (b), and AC impedance of the SFEG membrane after treating in the organic electrolyte for 9 days.

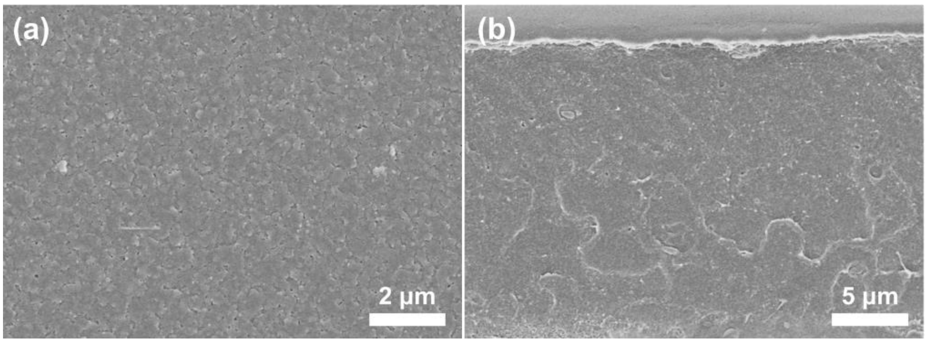

Figure S3. (a) Surface and (b) cross-sectional morphologies of SFEG membranes after heat treatment at 125°C for 1 h.

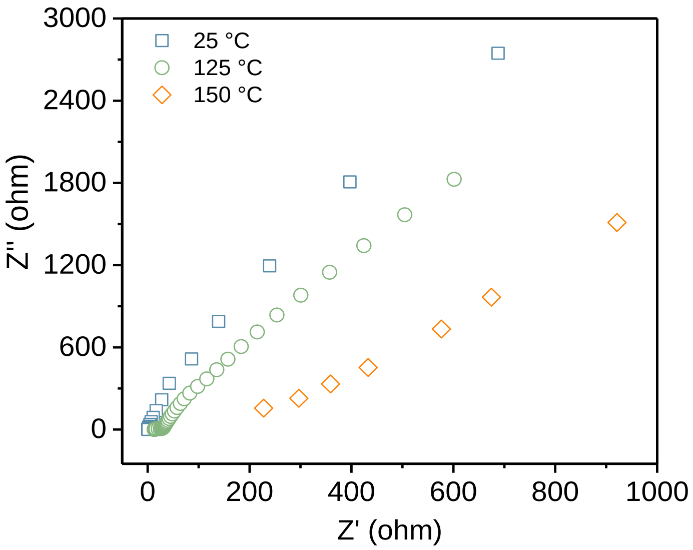

Figure S4. AC impedance of the SFEG membranes treated at various temperatures.

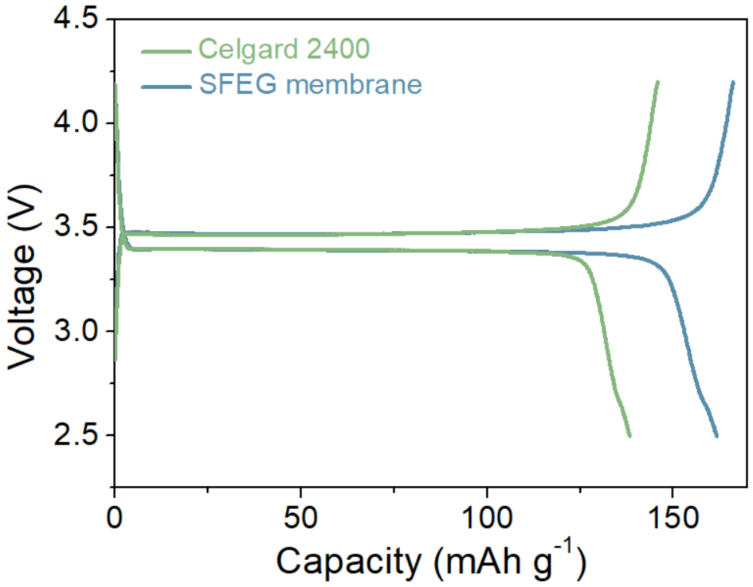

Figure S5. First charge-discharge capacities of the cells assembled with different separators.

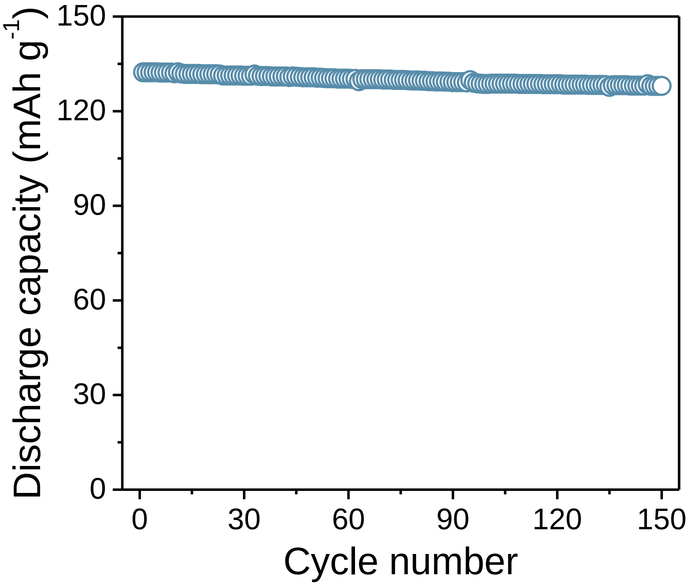

Figure S6. Long-cycle discharge capacity of LIBs with SFEG membranes at 1C.

Table S1. Fabrication methods of polymeric separators for LIBs.

Fabrication method	Preparation process
**Electrospinning**	1.Polymer is dissolved in organic solvent to prepare solution with a certain of concentration.<br>2.The polymer solution is electrospun at high voltage with appropriate spinning distance and feed rate.<br>3.Solvent, concentration, voltage and distance are considerably important factors for the electrospinning method.<br>4.The electrospinning process is carried at a fixed temperature and humidity.<br>5.Additionally, thermal treatment or mechanical pressing is carried out to increase the mechanical and stability of membrane.
**Electrophoretic deposition**	1.Polymer is dissolved in organic solvent to prepare solution.<br>2.The polymer solution is deposited in a substrate at electric field and graphite sheet for electric field control.<br>3.After amount of deposition time, the substrate is dried in ambient airflow.<br>4.Solvent, concentration and voltage are considerably important factors for the electrophoretic deposition.
**Thermally induced phase separation**	1.Polymer is dissolved in organic solvent to prepare solution.<br>2.An alternative temperature gradient induced by a non-uniform cooling upon the polymer solution to construct controlled pore architectures.<br>3.Solvent, concentration and temperature gradient are the key factors for the structure of separator.
**Phase inversion**	1.Polymer is dissolved in organic solvent to prepare solution.<br>2.The polymer solution is cast on substrate using coating machine, and dried at room temperature.
**Solution casting**	1.Polymer is dissolved in organic solvent to prepare solution.<br>2.The solution is cast onto a clean glass plate and quickly transferred to a constant climate chamber, kept fixed temperature and humidity.<br>3.After a time, the porous membrane is peeled off from the glass and thoroughly washed with water to remove solvent.<br>4.The resultant membrane is dried in the oven at high temperature.
**Casting through vacuum filtration**	1.Polymer is dispersed in solution to form suspension.<br>2.Suspension is poured onto the surface of a membrane under vacuum filtration to form film.<br>3.The film is thoroughly rinsed with deionized water, and dried at high temperature for some time under ambient atmosphere.
